# The development and validation of the Chinese safety climate scale using the item-response theory approach

**DOI:** 10.3389/fpsyg.2023.1119928

**Published:** 2023-07-10

**Authors:** Xiaokang Lyu, Binlin Zhao, Tingting Yang, Li Zhao

**Affiliations:** ^1^Department of Social Psychology, Zhou Enlai School of Government, Nankai University, Tianjin, China; ^2^Computational Social Science Laboratory, Nankai University, Tianjin, China; ^3^The Laboratory of Behavioral Economics and Policy Simulation, Nankai University, Tianjin, China; ^4^Shanghai International Theme Park Co., Ltd., Shanghai, China; ^5^Faculty of Economics and Management, East China Normal University, Shanghai, China

**Keywords:** safety climate, Chinese safety climate scale, item response theory, group-level, organization-level

## Abstract

**Introduction:**

To develop a valid and practical tool to measure the safety climate in China, and further raise awareness of it in Chinese industries, we developed the Chinese safety climate scale (including two subscales at the levels of organization and group separately) based on the work of Huang et al. in 2017.

**Methods:**

A descriptive survey with the convenience sampling method was conducted in Shanghai Disney Resort, China. A sample of 1,570 employees was involved in the final data analyses. The item response theory (IRT) analyses with graded response models were conducted using.

**Results:**

The unidimensionality and local independence assumption were held. The Cronbach’s α of organization- and group- level safety climate subscale was 0.912 and 0.937, respectively. The confirmatory factor analysis (CFA) showed good model fit for a one-factor model of the organization-level safety climate subscale, χ^2^ (*df* = 20) = 129.158, *p* < 0.001, CFI = 0.994, TLI = 0.992, NFI = 0.993, IFI = 0.994, RMSEA = 0.059, 90% CI = (0.050, 0.069), and SRMR = 0.048. A one-factor model also fits well for the group-level safety climate subscale, χ^2^ (*df* = 44) = 219.727, *p* < 0.001, CFI = 0.996, TLI = 0.9925, NFI = 0.995, IFI = 0.996, RMSEA = 0.050, 90% CI = (0.044, 0.057), and SRMR = 0.046. Discrimination and difficulty parameters showed that all items effectively spanned the range of the latent trait and could successfully separate participants at different safety climate levels. Items in the organization-level and group-level Chinese safety climate subscales had significantly different discrimination parameters, fitted well with the models, and had a substantive relationship with the latent traits.

**Discussion:**

The Chinese safety climate scale was reliable and valid overall. They can facilitate the research and survey regarding the safety climate in China.

## 1. Introduction

Safety climate refers to how employees collectively perceive the policies, regulations, and practices of the organization regarding the value and significance of safety (Zohar, 1980; Huang et al., 2017; Luo, 2020). Existing literature illustrates that safety climate is crucial for implementing a safety management system by impacting employees’ attitudes toward safety, how they perform their work, and how they interact with one another (Kim et al., 2019; Omidi et al., 2022; Ricci et al., 2022). Employees who are more concerned with safety and who have a sufficient understanding of safe operation perform more standardly in the work process, which in turn lowers the probability of accidents and protects the safety of themselves and other employees.

The investigation of safety climate is also important in China. “Safety first” is an important principle for Chinese industries to follow when conducting manufacturing. The acceleration of industrialization, the enhancement of social management systems, and the amplification of public supervision have made safety the top priority in recent years. In management practice, industries typically “hardware invest” in their employees with standard technical methodologies, tools, and procedures. In other words, industries attach importance to the construction and upgrading of physical entities to improve safety. For example, in an amusement facility manufacturing industry, the continuous improvement of the hardware logic control reliability technology in the amusement facility system and the use of a large number of sophisticated artificial intelligence sensor technology have continuously improved the intrinsic safety reliability of amusement equipment. However, safety incidents still happen as the diminishing marginal benefits of “hardware investment.” The reason might be the lack of “software investment,” such as reciprocal care, information sharing, and regular communication. In other words, industries can enhance the safety level by improving soft power, such as taking care of employees’ mental feelings, sharing safety information, and increasing confidence in the safety of the industry. To address this issue, the focus of industries should be expanded from “explicit factors” such as work equipment to “implicit factors” such as individual perception and organizational climate during the economic take-off in recent years in China. One of the most important “software investments” that industries are paying attention to is safety climate.

To better understand the characteristics of safety climate, the factors that can influence the safety climate and possible impact mechanisms, a valid tool is needed to measure the safety climate. To measure the safety climate, Zohar conducted a quantitative investigation in 20 industrial organizations to understand the meaning of safety climate, and developed a 40-item safety climate scale, which mainly measured the organizational safety climate (Zohar, 1980). To measure the safety climate more comprehensively, Zohar (2000) further developed the concept of group safety climate and conducted a quantitative study in 53 work groups within a manufacturing company. After that, Zohar and Luria (2005) proposed the two-level structure of safety climate and developed a 16-item safety climate subscale for each level. There are two levels within the structure of safety climate (Zohar and Luria, 2005; Huang et al., 2017). The first level is the group-level safety climate, which refers to the perceptions of the employees’ direct supervisors’ commitment to and emphasis on safety. It is possible to consider safety climate as a psychological construct, and the corresponding perceptions can be complied at the group level (Kao et al., 2021). Psychological safety climate, as the foundation for safety climate at higher levels, may contribute to the development of enterprises. The second level is the organization-level safety climate, which is how employees perceive the organization’s commitment to and emphasis on safety. The safety climate at the group level is positively influenced by the organization-level safety climate, and it can positively influence employees’ tendency toward safety behavior (Zohar and Luria, 2005; Newaz et al., 2019; Luo, 2020). It has been found that safety climate significantly impacts the safety performance in small and medium enterprises (Asad et al., 2022).

To improve the utility of the safety climate scale, Huang et al. (2017) shortened them by conducting an item response theory (IRT) analysis with a sample of 29,179 industry employees using graded response models in two different methods. The first method was selecting the above-average discriminating ability items. The second method was choosing the most informative items that collectively maintain at least 30% of the information from the original scale. Based on the first way, a shortened version of the safety climate scale with eight items at the organization level and 11 items at the group level was obtained. Based on the second way, a further shortened version of the safety climate scale with four items at the organization level and four items at the group level was obtained.

However, there are few suitable and specialized scales for measuring the safety climate in China. For example, Li et al. (2023) proposed a study to investigate the improvement of the safety climate in high-risk Chinese industries, however, they used the manager’s commitment to safety to represent the safety climate. The lack of a practical tool to measure the safety climate makes it difficult to perform a precise survey to enhance industrial safety in China. To bring industries’ attention to safety climate and to increase the usefulness of the safety climate scale, we constructed the Chinese (to be more specific, Mandarin) version of the safety climate scale based on Huang et al.’s (2017) work and validated them in this study. The native language helps participants understand the questions accurately and provide precise answers when completing the survey. Then, meaningful data can be obtained by researchers and accurate conclusions can be drawn. Only when the data and conclusions are accurate, they can contribute to the corresponding research area. The safety climate scale created by Huang et al. (2017) was published publicly with open access under the CC BY license. We did not investigate the preliminary questionnaire created by Zohar and Luria (2005) due to Huang et al.’s (2017) safety climate scale had been proven to be reliable and valid. It contained fewer items than Zohar and Luria’s (2005) preliminary safety climate scale, so it could be more widely used in research and practice. For the similar aim that we would like to develop the Chinese version of the safety climate scale to facilitate the investigation and measurement in China, it would be better to use a shortened version. However, given that (1) more characteristics could be retained in the 8-item organization-level and 11-item group-level safety climate subscales. For example, in comparison with the 4-item organization-level subscale, the 8-item version contained questions regarding the individual safety rights of workers and the balance between work speed, industrial efficiency, and safety. In comparison with the 4-item group-level subscale, the 11-item version contained more details about safety in different scenarios, such as “under pressure,” “when fixing equipment or machines” and “at the end of the shift”; and (2) the total test information, which was the sum of the information an item contributed, retained from the original scale of the 8-item organization-level and 11-item group-level safety climate subscales was 56.94 and 77.71%, which were higher than that of the 4-item organization-level (30.30%) and 4-item group-level (30.89%) safety climate subscales. Therefore, the safety climate scale with the 8-item organization-level subscale and the 11-item group-level subscale in Huang et al. (2017) was used as the prototype of the scale translation.

To test whether the translated Chinese safety climate scale could be used as a valid instrument to measure the safety climate in China, the item response theory (IRT) analyses was conducted. IRT is a probabilistic non-linear modeling method and it is commonly used to create and assess scales measuring psychological features (Embretson and Reise, 2013; Chiesi et al., 2020). It analyzes each scale item’s capacity to differentiate respondents and calculates the likelihood that particular response options of each item endorsed by respondents (Huang et al., 2017). IRT, in other words, explores the relationship between the latent trait of participants and item responses by the item option response functions (ORFs). The latent trait of participants and the characteristics of items influence the likelihood that an individual would react to a certain item, and as the degree of the latent trait increases, the likelihood of choosing a higher response option increases (Samejima, 2016). In addition, in comparison with the classic theory test analyses, which focus more on the scale’s composite score, IRT takes the information at the item level, such as each item’s difficulty and its capacity for discriminating, without the dependence on the sample (Crocker and Algina, 1986; Ye et al., 2018). Therefore, when developing an evaluation instrument, IRT analysis can provide more details on the factor structure and internal consistency, and it is appropriate to assess the validity of measurement scales.

To sum up, researchers and investigators are short of an effective instrument to measure the safety climate in Chinese industries currently. In order to promote safety climate research and draw the attention of industries to safety climate in China, the current study aims to create a reliable and valid Chinese safety climate scale at both levels of organization and group using the item-response theory approach.

## 2. Materials and methods

### 2.1. Study design

A descriptive survey was conducted in this study for the purpose of developing a Chinese safety climate scale. The items were generated based on the safety climate scale in Huang et al. (2017). Then the translated version of Chinese safety climate scale was tested on employees in a Chinese company. Empirical data were collected and analyzed to evaluate the reliability and validity of the Chinese safety climate scale using the item-response theory approach.

### 2.2. Participants

Survey data were collected anonymously from 1,642 employees in Shanghai Disney Resort, China, from January to July 2020 with a convenience sampling method. Seventy-two participants were excluded due to either being under 18 years of age or not passing the validation questions. Two validation questions, including the participants’ education level and the number of years of employment, were used. They were asked at the end of the questionnaire. If at least one of the answers to these two validation questions was different from that in the section of demographic information questions, we marked the corresponding questionnaire and excluded it from the following data analyses. For example, if an individual’s answer for the number of years of employment was 1 in the section of demographic information questions, but it was 2 in the section of validation questions, this individual would be excluded from the following data analyses. Data from 1,570 participants were included in the final analyses. The response rate is 95.62%.

Participants provided information regarding their age, gender, education level, and the number of years working in Shanghai Disney Resort in the survey. The demographic characteristics of participants are displayed in Table 1.

**TABLE 1:** Demographic characteristics of participants (*N* = 1,570).

Variable		*M*	*SD*	*Min*	*Max*
Age		32.07	8.93	18	60
		***N***		**%**	
Gender	Male	510		32.48	
	Female	1060		67.52	
Education level	High school and below	675		43.00	
	High vocational school or junior college	629		40.06	
	Undergraduate college	257		16.37	
	Master’s degree and above	9		0.57	
Years of working	1	245		15.61	
	2	203		12.93	
	3	431		27.45	
	4	675		42.99	
	5	16		1.02	

### 2.3. Measures

The Chinese version of the safety climate scale was developed based on Huang et al.’s (2017) 8-item organization-level and 11-item group-level English version of safety climate subscales. At the organization level, employees were asked question items regarding the top management at the company, such as “Uses any available information to improve existing safety rules,” “Tries to continually improve safety levels in each department,” and “Requires each manager to help improve safety in his or her department.” At the group level, employees were asked question items regarding the direct supervisor, such as “Discusses how to improve safety with us,” “reminds workers who need reminders to work safely,” and “Uses explanations (not just compliance) to get us to act safely.” The items scored on a 5-point Likert scale from 1 (completely disagree) to 5 (completely agree). The Huang et al.’s safety climate scale was shown in Appendix A. The forward-backward translation was conducted, and then a group of experts critically evaluated the Chinese scale (Zhao et al., 2022). A psychologist and a safety management expert with advanced English proficiency were recruited to translate the English version of the scale into Chinese. Without reading the original English scale, two professors in the Department of English at Nankai University translated the scale from Chinese into English, which was not significantly different from the original version. Finally, the scale was evaluated and modified by a group of six other psychologists and safety management experts in order to address potential problems, such as ambiguity, difficulty in comprehension, and lack of conciseness. To ensure the reliability of the translated scale, before being used in the formal study, it was tested with a Chinese sample of 300, who were employees (Age: *M* = 32.97, *SD* = 9.37; Year of working: *M* = 3.10, *SD* = 1.10) from Tianjin Fantawild Adventure. The snowball sampling method was applied in recruiting participants. Tianjin Fantawild Adventure was the same type of company as Shanghai Disney Resort. The Cronbach’s α value was 0.925 for the Chinese organization-level and 0.944 for the Chinese group-level safety climate subscales, respectively. Additionally, the Chinese safety climate scale was developed on the basis of Huang et al.’s (2017) safety climate scale, which were the short version of the preliminary safety climate scale created by Zohar and Luria (2005). In Zohar and Luria’s (2005) safety climate scale, there were 16 items for the organization level and 16 items for the group level. For the aim of increasing the utility of safety climate scale, explore the emergence of safety climate, and factors that could impact the safety climate, Huang et al. (2017) shortened the number of items in the scale, but they devoted to maximize the information included in the scale. The 8-item organization-level and 11-item group-level safety climate subscales retained 56.94 and 77.71% of the total test information of Zohar and Luria’s (2005) scale, respectively. The retained items in the 8-item organization-level subscale were items 11, 3, 9, 14, 16, 12, 6, and 13. The retained items in the 11-item group-level subscale were items 10, 4, 3, 9, 5, 13, 6, 2, 14, 11, and 15. (Please see Huang et al. (2017) for details.) Therefore, the items were reasonable for relevant conceptual measures of safety climate. The final version of the Chinese safety climate scale is in Appendix A.

### 2.4. Procedure

Reference number IRB: NKUIRB2020103 was obtained after the Institutional Review Board of Nankai University gave ethical approval for the study to be conducted on 1/13/2020. All questionnaires were distributed online by using Wenjuanxing^1^, which is a professional survey website in China. To be more specific, we created the questionnaire, which contained a brief introduction, a consent form, demographic information questions, and the Chinese safety climate scale, on Wenjuanxing. Then the survey link for this questionnaire created by Wenjuanxing was distributed to all department managers of the Shanghai Disney Resort industry. Then the department managers sent the survey link to all workers in their department. To avoid possible ethical issues, the department managers participated in tutorials regarding volition, privacy, confidentiality, informed consent, and anonymity. Following the principles of the Declaration of Helsinki, informed consent was obtained from participants. Especially, they were made fully aware that they could stop participating in the survey at any time. Participants volunteered to fill out the questionnaire. The data collected in this study were stored securely and anonymously, and we would not use them for purposes other than scientific research.

### 2.5. Data analysis procedure

#### 2.5.1. Reliability test

To determine the reliability of the Chinese safety climate scale, the values of Cronbach’s α and McDonald’s omega for the Chinese organization- and group- level safety climate subscales were calculated. The widely accepted standard for good internal consistency (Cronbach’s α = 0.70 and McDonald’s omega = 0.70) was applied (see Taber, 2018; Kim et al., 2022).

#### 2.5.2. Local independence

The residual correlations between items were used to test the local independence, which states that a response to one item is unrelated to a response to any other item. Additionally, items with residual correlations above 0.2 should be removed from the analysis (Cantó-Cerdán et al., 2021).

#### 2.5.3. IRT analysis

Considering that the safety climate scales developed by Zohar and Luria (2005) and Huang et al. (2017) were multidimensional (including the organization level and the group level) in nature, a multidimensional graded response model could be applied, which means the 2-factor confirmatory factor analysis (CFA) could be performed. Based on the model fitting indices, whether the organization level and the group level should be treated separately could be further considered.

To assess the IRT model-data fit, for the whole Chinese safety climate scale, at the model-level, a unidimensional graded response model and a 2-factor graded response model could be built and compared. In order to determine which model fits better, a comparison of the change in −2*loglikelihood (−2*LL, which is based on a Chi-square distribution), AIC, and BIC can be evaluated. The result of the likelihood ratio test can indicate either the unidimensional model or the 2-factor model fits better. Presumably, the 2-factor model should fit better since the Chinese safety climate scale contained two levels (i.e., organization and group) of measurement.

Each item’s degree of model fit was evaluated using the index S-χ^2^. RMSEA values less than 0.06 were considered evidence of adequate fit (Vilca et al., 2022). The Chinese safety climate scale at the levels of organization and group were all based on five response options. Therefore, the graded response model estimates one slope (also known as item discrimination) parameter and four difficulty (also known as latent trait value) parameters for each item of the scale by the item option response functions (ORFs). The slope parameter, which is commonly represented by the Greek letter alpha (α), shows how well the items discriminate against different levels of the latent trait. The difficulty parameter, which is commonly represented by *b*, indicates the threshold at which a respondent with a particular latent trait has an equal likelihood of endorsing a particular item. Information, which could reflect the amount of empirical information each item could contribute to the entire scale (Toland, 2014), contained by each item and the scale was also calculated. Factor loadings, which represented the degree to which an item and a latent variable were correlated were also calculated to evaluate the scale.

Furthermore, to better visually examine the item and scale characteristics in Chinese safety climate scale at organization and group levels, various plots were drawn to display how each item and the overall scale relate to the latent trait across trait values.

The software used in this study were JASP 0.16.3 (JASP Team, 2022) and R packages, including *EFA.dimensions*, *ltm*, and *mirt*.

#### 2.5.4. Criterion related validity test

The criterion related validity test was conducted to better reflect the purpose of the Chinese safety climate scale and demonstrate safety climate as a root construct in safety management studies. Safety behavior tendency, and safety rules attitude were measured. The safety behavior tendency scale created by Li et al. (2018) was used to measure the safety behavior tendency in this study. The safety rules attitude was measured the attitude to safety rules scale, which was created by Auzoult and Ngueutsa (2019). The correlations between safety climate, safety behavior tendency, and safety rules attitude were calculated.

## 3. Results

### 3.1. Preliminary analyses

The mean organization- and group- level scores for the Chinese safety climate subscales were 4.32 (*SD* = 0.63) and 4.39 (*SD* = 0.61), respectively. Table 2 showed the frequency and percentage of respondents who endorsed a given choice on a 5-point Likert scale for each item (i.e., the option endorsement frequency and percentage), mean and standard deviation for each item of the Chinese organization- and group -level safety climate subscales, respectively.

**TABLE 2:** Basic descriptive information for Chinese organization-level safety climate (OSC) and group-level safety climate (GSC) subscales (*N* = 1,570).

Scale	Item	Option 1		Option 2		Option 3		Option 4		Option 5		*Mean*	*SD*
		***F***	***P***	***F***	***P***	***F***	***P***	***F***	***P***	***F***	***P***		
OSC	1	13	0.83%	26	1.66% (2.49%)	297	18.92%	617	39.30%	617	39.30%	4.15	0.84
	2	18	1.15%	32	2.04% (3.19%)	292	18.60%	569	36.24%	659	41.97%	4.16	0.88
	3	14	0.89%	15	0.96% (1.85%)	222	14.14%	575	36.62%	744	47.39%	4.29	0.81
	4	15	0.96%	20	1.27% (2.23%)	209	13.31%	567	36.11%	759	48.34%	4.30	0.82
	5	18	1.15%	23	1.46% (2.61%)	147	9.36%	582	37.07%	800	50.96%	4.35	0.80
	6	15	0.96%	16	1.02% (1.98%)	101	6.43%	514	32.74%	924	58.85%	4.48	0.74
	7	12	0.76%	11	0.70% (1.46%)	81	5.16%	461	29.36%	1005	64.01%	4.55	0.70
	8	15	0.96%	13	0.83% (1.79%)	194	12.36%	619	39.43%	729	46.43%	4.30	0.79
GSC	1	10	0.64%	21	1.34% (1.98%)	120	7.64%	556	35.41%	863	54.97%	4.43	0.74
	2	13	0.83%	23	1.46% (2.29%)	180	11.46%	625	39.81%	729	46.43%	4.30	0.79
	3	18	1.15%	23	1.46% (2.61%)	151	9.62%	552	35.16%	826	52.61%	4.37	0.81
	4	15	0.96%	33	2.10% (3.06%)	182	11.59%	559	35.61%	781	49.75%	4.31	0.83
	5	13	0.83%	12	0.76% (1.59%)	91	5.80%	506	32.23%	948	60.38%	4.51	0.72
	6	9	0.57%	10	0.64% (1.21%)	79	5.03%	487	31.02%	985	62.74%	4.55	0.68
	7	11	0.70%	14	0.89% (1.59%)	85	5.41%	483	30.76%	977	62.23%	4.53	0.70
	8	8	0.51%	11	0.70% (1.21%)	84	5.35%	391	24.90%	1076	68.54%	4.60	0.67
	9	19	1.21%	28	1.78% (2.99%)	138	8.79%	534	34.01%	851	54.20%	4.38	0.82
	10	12	0.76%	23	1.46% (2.22%)	130	8.28%	599	38.15%	806	51.34%	4.38	0.76
	11	41	2.61%	57	3.63% (6.24%)	339	21.59%	611	38.92%	522	33.25%	3.97	0.96

Frequency (*F*) means the number of respondents who endorsed specified options 1–5 on a 5-point Likert scale for each item (Option 1 = completely disagree, Option 5 = completely agree). Percentage (*P*) represents the percentage of respondents who endorsed the corresponding option. Values in parenthesis represent response percentages after collapsing the first two response options into one category.

The confirmatory factor analysis (CFA) conducted with JASP 0.16.3 showed good model fit for a 2-factor model of the Chinese safety climate scale, χ^2^ (*df* = 151) = 1770.081, χ^2^/*df* = 11.72, *p* < 0.001, CFI = 0.994, TLI = 0.994, NFI = 0.994, IFI = 0.994, RMSEA = 0.083, 90% CI = (0.079, 0.086), and SRMR = 0.053. Furthermore, if the organization-level and group-level Chinese safety climate subscales were treated separately, a one-factor model fit well for the organization-level safety climate subscale, χ^2^ (*df* = 20) = 129.158, *p* < 0.001, CFI = 0.994, TLI = 0.992, NFI = 0.993, IFI = 0.994, RMSEA = 0.059, 90% CI = (0.050, 0.069), and SRMR = 0.048. In the exploratory factor analysis, the ratio of the first and second unrotated components’ eigenvalues was 6.34, which was a good sign of unidimensionality. A one-factor model also fit well for the group-level safety climate subscale, χ^2^ (*df* = 44) = 219.727, *p* < 0.001, CFI = 0.996, TLI = 0.9925 NFI = 0.995, IFI = 0.996, RMSEA = 0.050, 90% CI = (0.044, 0.057), and SRMR = 0.046. In the exploratory factor analysis, the ratio of the eigenvalues of the first and second unrotated components was 8.32, which was a good indicator of unidimensionality.

The Cronbach’s α of the Chinese organization- and group -level safety climate subscales was 0.912 with 95% CI (0.899, 0.922) and 0.937 with 95% CI (0.929, 0.945), respectively. The McDonald’s omega of the Chinese organization- and group -level safety climate subscales was 0.912 with 95% CI (0.905, 0.918) and 0.936 with 95% CI (0.931, 0.941), respectively.

By using the *EFA.dimensions* package in R, it was found that for the Chinese organization-level safety climate subscale, the number of residual correlations > = 0.1 is 2 and the number of residual correlations > = 0.2 is 1; For the Chinese group-level safety climate subscale, the number of residual correlations > = 0.1 is 9 and the number of residual correlations > = 0.2 is 3. The assumption of local independence was satisfied because no high residual correlations (> 0.2) were found among the eight items at the Chinese organization-level and 11 items in the Chinese group-level safety climate subscales.

### 3.2. IRT model results

#### 3.2.1. IRT model testing

IRT analyses were performed with R. The Latent Trait Modeling (*ltm*) package developed by Rizopoulos (2007) and the Multidimensional Item Response Theory (*mirt*) package developed by Chalmers (2012) were used.

By considering the organization level and group level of the safety climate scale together, the unidimensional model yielded a −2*LL value of −22,308.21, AIC = 44,806.41, BIC = 45,315.5, whereas the 2-factor model resulted in a −2*LL value of −21,510.09, AIC = 43,246.18, BIC = 43,851.72. The likelihood ratio test yielded an LRT = 1,596.24, *df* = 18, *p* < 0.001. This indicates that the 2-factor model was significantly better than the unidimensional model, and the Chinese safety climate scale items had significantly different discrimination parameters.

#### 3.2.2. IRT parameters and information

Results for the parameter of the 2-factor model for the Chinese safety climate scale items are displayed in Table 3. The index S-χ^2^ was used to assess how well each item fitted the model. All RMSEA values for the Chinese organization- and group- level safety climate subscales were less than 0.06, indicating that the items had an adequate fit with the model.

**TABLE 3:** Results of parameters and information of the 2-factor graded response models for Chinese safety climate scale items.

Item	Slope_1_ (se)	Slope_2_ (se)	Difficulty				S-χ^2^	*df*	RMSEA-χ^2^	*p*	FL_1_	FL_2_
			**Diff_1_ (se)**	**Diff_2_ (se)**	**Diff_3_ (se)**	**Diff_4_ (se)**						
OSC1	−1.77 (0.10)	–	6.89 (0.37)	5.34 (0.23)	2.00 (0.11)	−0.68 (0.10)	104.76	64	0.020	0.001	0.75	–
OSC2	−2.12 (0.12)	–	6.94 (0.36)	5.40 (0.23)	2.15 (0.11)	−0.56 (0.12)	104.92	73	0.017	0.009	0.86	–
OSC3	−3.17 (0.24)	–	10.27 (0.64)	8.54 (0.50)	3.69 (0.27)	−0.30 (0.16)	127.34	57	0.028	0	0.89	–
OSC4	−3.00 (0.31)	–	9.73 (0.65)	7.98 (0.53)	3.69 (0.29)	−0.22 (0.14)	110.44	56	0.025	0	0.85	–
OSC5	−2.10 (0.10)	–	8.29 (0.43)	6.60 (0.30)	3.72 (0.17)	0.02 (0.10)	84.50	54	0.019	0.005	0.59	–
OSC6	−2.31 (0.11)	–	9.34 (0.50)	7.66 (0.36)	4.67 (0.20)	0.69 (0.11)	46.28	47	< 0.001	0.502	0.60	–
OSC7	−2.56 (0.13)	–	11.14 (0.66)	9.31 (0.50)	5.68 (0.26)	1.28 (0.13)	56.78	43	0.014	0.078	0.58	–
OSC8	−1.59 (0.08)	–	7.36 (0.39)	6.24 (0.28)	2.91 (0.12)	−0.25 (0.08)	142.67	57	0.031	0	0.50	–
GSC1	–	−2.06 (0.11)	9.36 (0.55)	6.96 (0.32)	4.04 (0.16)	0.33 (0.09)	59.22	50	0.011	0.174	–	0.57
GSC2	–	−2.27 (0.12)	8.94 (0.49)	6.85 (0.30)	3.43 (0.14)	−0.34 (0.08)	87.75	51	0.021	0.001	–	0.67
GSC3	–	−2.08 (0.11)	7.87 (0.39)	6.34 (0.27)	3.51 (0.14)	0.16 (0.08)	75.41	55	0.015	0.035	–	0.63
GSC4	–	−2.18 (0.12)	8.30 (0.44)	6.17 (0.26)	3.25 (0.14)	−0.05 (0.08)	96.32	56	0.021	0.001	–	0.66
GSC5	–	−3.26 (0.18)	10.96 (0.64)	9.22 (0.48)	5.62 (0.25)	0.98 (0.12)	56.21	44	0.013	0.103	–	0.85
GSC6	–	−4.73 (0.44)	16.82 (1.47)	13.55 (1.12)	8.08 (0.63)	1.65 (0.21)	40.09	39	0.004	0.422	–	0.90
GSC7	–	−3.94 (0.22)	13.60 (0.83)	10.95 (0.58)	6.78 (0.32)	1.39 (0.14)	37.01	42	< 0.001	0.69	–	0.87
GSC8	–	−3.28 (0.22)	12.48 (0.88)	9.79 (0.60)	5.86 (0.29)	1.83 (0.14)	47.30	45	0.006	0.379	–	0.85
GSC9	–	−2.47 (0.18)	7.50 (0.43)	6.02 (0.32)	3.58 (0.19)	0.32 (0.10)	93.09	71	0.014	0.041	–	0.90
GSC10	–	−3.18 (0.21)	10.17 (0.61)	7.83 (0.42)	4.53 (0.22)	0.12 (0.12)	82.12	54	0.018	0.008	–	0.94
GSC11	–	−1.60 (0.12)	4.75 (0.22)	3.68 (0.17)	1.43 (0.10)	−0.94 (0.08)	132.03	88	0.018	0.002	–	0.84

OSC, organization-level safety climate; OSC1-OSC8 refer to the 8 items in the Chinese version of the OSC subscale. GSC, group-level safety climate; GSC1-GSC11 refer to the 11 items in the Chinese version of the GSC subscale.

For the eight Chinese organization-level safety climate items, the range of the slope/discrimination parameters was from −3.17 (item 3) to −1.59 (item 8), and for the 11 Chinese group-level safety climate items, the range of the slope/discrimination parameters was from −4.73 (item 6) to −1.60 (item 11). The negative slope values could be considered problematic as it would mean that items with increasing levels of ability were less likely to endorse more severe response options.

Although the organization-level safety climate and the group-level safety climate could be treated as two factors in the Chinese safety climate scale, due to the discrimination values were negative in the 2-factor model, we continued to conduct the IRT analyses with treating the organization-level and group-level safety climate subscales separately. Results for the parameter and information of the separate unidimensional models for the items in OSC and GSC subscales are displayed in Table 4 and Table 5. For the eight Chinese organization-level safety climate items, the range of the discrimination parameters was from 1.83 (item 1) to 3.53 (item 7), suggesting good discrimination (slope parameter greater than 0.50, Srem-Sai et al., 2022). The percentage of total test information each item provided ranged from 8.14 to 17.20%. Item 7 had the strongest relationship with the latent trait and measured organization-level safety climate more precisely than other items. The difficulty parameters reflected a sizable range of the underlying construct, −3.717 (item 1, diff1) to 0.333 (item 1, diff4), indicating that the Chinese organization-level safety climate subscale was generally more useful in identifying respondents with low to average organization safety climate scores than very high organization safety climate scores.

**TABLE 4:** Results of parameters and information of the full graded response models for OSC scale items.

Item	Slope	Difficulty				S-χ^2^	*df*	RMSEA-χ^2^	*p*	Factor loading	Information total test = 67.44	
		**Diff1**	**Diff2**	**Diff3**	**Diff4**						**Value (Rank)**	**Percentage**
OSC1	1.833	−3.717	−2.896	−1.100	0.333	82.91	34	0.030	< 0.001	0.74	5.49 (8)	8.14%
OSC2	1.932	−3.399	−2.635	−1.056	0.242	69.79	34	0.026	< 0.001	0.76	5.67 (7)	8.41%
OSC3	3.009	−3.148	−2.625	−1.141	0.053	56.63	22	0.032	< 0.001	0.87	9.78 (4)	14.50%
OSC4	3.022	−3.054	−2.518	−1.172	0.031	48.93	22	0.028	0.001	0.87	9.79 (3)	14.51%
OSC5	2.836	−3.015	−2.436	−1.390	−0.050	54.06	26	0.026	0.001	0.86	8.90 (5)	13.19%
OSC6	3.158	−3.076	−2.558	−1.575	−0.268	35.28	23	0.018	0.049	0.88	10.06 (2)	14.91%
OSC7	3.526	−3.181	−2.688	−1.677	−0.410	50.69	19	0.033	< 0.001	0.90	11.60 (1)	17.20%
OSC8	2.102	−3.435	−2.945	−1.401	0.082	76.54	28	0.033	< 0.001	0.78	6.16 (6)	9.13%

OSC, organization-level safety climate; OSC1-OSC8 refer to the 8 items in the Chinese version of the OSC scale.

**TABLE 5:** Results of parameters and information of the full graded response models for GSC scale items.

Item	Slope	Difficulty				S-χ^2^	*df*	RMSEA-χ^2^	*p*	Factor loading	Information total test = 106.76	
		**Diff1**	**Diff2**	**Diff3**	**Diff4**						**Value (Rank)**	**Percentage**
GSC1	2.398	−3.520	−2.695	−1.564	−0.091	72.07	36	0.025	< 0.001	0.83	7.56 (8)	7.08%
GSC2	2.659	−3.263	−2.534	−1.239	0.165	58.07	35	0.020	0.008	0.85	8.69 (6)	3.27%
GSC3	2.435	−3.104	−2.525	−1.379	−0.028	99.71	41	0.030	< 0.001	0.83	7.29 (9)	6.09%
GSC4	2.531	−3.182	−2.390	−1.235	0.060	98.51	45	0.028	< 0.001	0.84	8.01 (7)	6.83%
GSC5	3.547	−3.094	−2.614	−1.572	−0.226	40.31	27	0.018	0.048	0.91	11.74 (3)	7.50%
GSC6	4.847	−3.195	−2.688	−1.589	−0.270	26.34	22	0.011	0.238	0.96	17.56 (1)	8.14%
GSC7	4.199	−3.120	−2.559	−1.580	−0.271	29.02	24	0.012	0.220	0.94	14.79 (2)	8.88%
GSC8	3.422	−3.415	−2.752	−1.640	−0.464	51.17	27	0.024	0.003	0.91	11.64 (4)	10.90%
GSC9	2.241	−3.139	−2.498	−1.448	−0.068	92.13	48	0.024	< 0.001	0.81	6.50 (10)	11.00%
GSC10	2.860	−3.262	−2.496	−1.401	0.027	61.48	35	0.022	0.004	0.87	9.48 (5)	13.85%
GSC11	1.340	−3.348	−2.570	−0.928	0.729	172.14	61	0.034	< 0.001	0.63	3.49 (11)	16.45%

GSC, group-level safety climate; GSC1-GSC11 refer to the 11 items in the Chinese version of the GSC scale.

Results for the 11 Chinese group-level safety climate items were quite similar. The discrimination parameters ranged from 1.34 (item 11) to 4.85 (item 6), suggesting good discrimination. The percentage of total test information each item provided ranged from 8.14 to 16.45%. Item 6 had the strongest relationship with the latent trait and measured group-level safety climate more precisely than other items. The difficulty parameters reflected a sizable range of the underlying construct, −3.520 (item 1, diff1) to 0.729 (item 11, diff4), indicating that the Chinese group-level safety climate subscale was generally more useful in identifying participants with low to average group-level safety climate scores than the very high level of safety climate.

Factor loadings for the organization- and group- level safety climate subscales, as shown in Tables 3–5, were all larger than 0.50, indicating all of the items had a substantive relationship with the latent trait.

**FIGURE 1:**
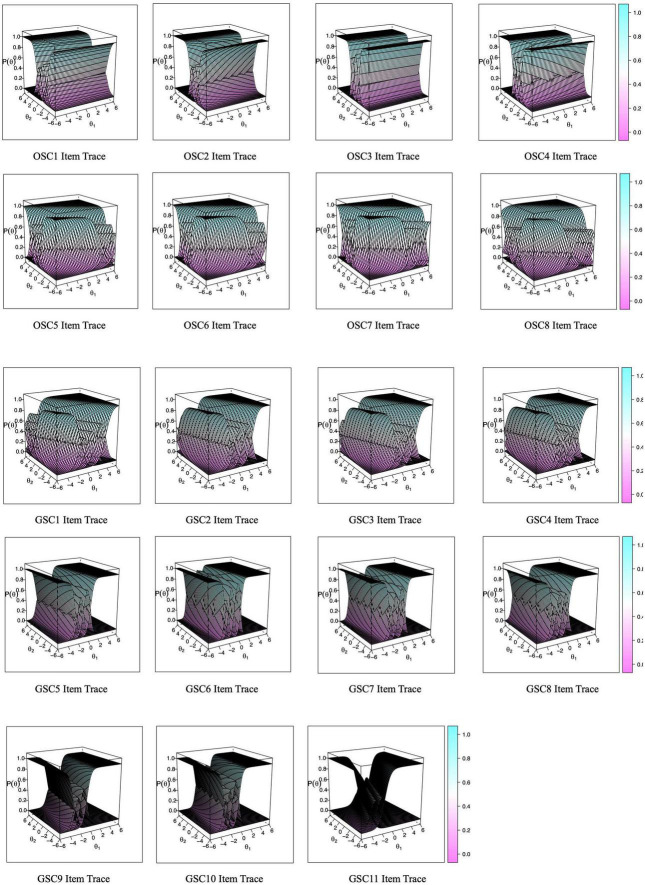
Item trace for the Chinese safety climate scale. OSC1-OSC8 refer to the eight items in the Chinese version of the OSC subscale. GSC1-GSC11 refer to the 11 items in the Chinese version of the GSC subscale.

**FIGURE 2:**
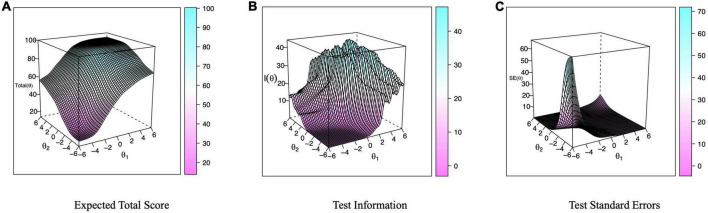
Expected total score, test information, and test standard errors for the Chinese safety climate scale.

**FIGURE 3:**
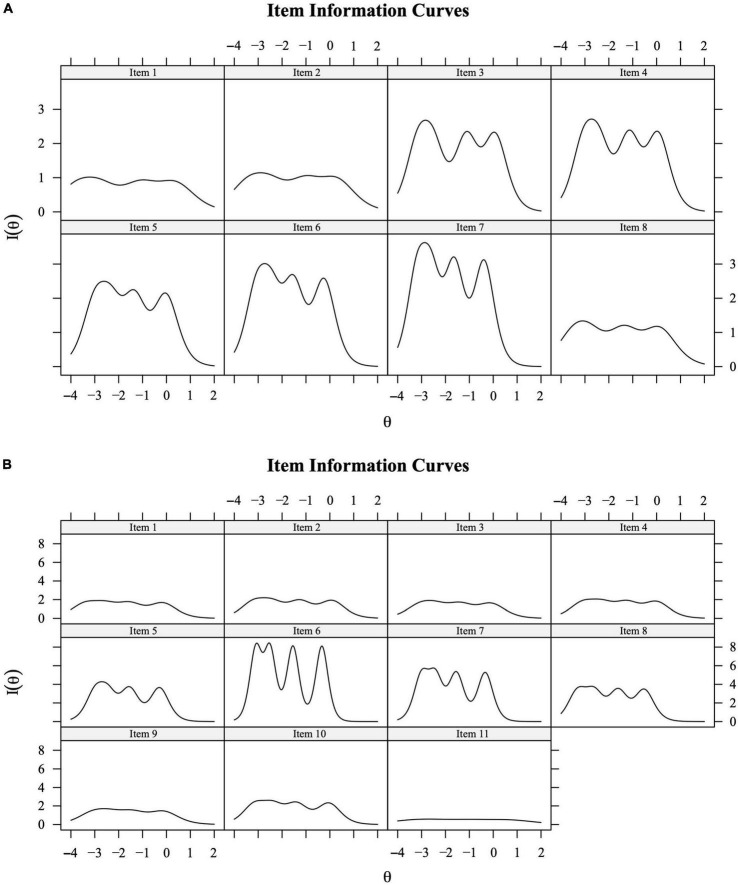
**(A)** Item Information Curve for Chinese organization-level safety climate subscale items. **(B)** Item Information Curve for Chinese group-level safety climate subscale items. *I* (θ) means information.

For the 2-factor model, the item trace plots for the items in the Chinese safety climate scale are shown in Figure 1. The expected total score, test information, and test standard errors are shown in Figure 2. The Chinese safety climate scale could provide optimal parameter evaluation with moderate safety climate levels. Again, since the values for the slope parameter were questionable in the 2-factor model, we continued to investigate the item and total information by separating the organization level and the group level. For the unidimensional models for the organization-level and group-level safety climate subscales, the item information curves, as shown in Figures 3A, B), depict how the item information of each item changed with the levels of θ of the Chinese organization- and group- level safety climate subscales, respectively. For the organization-level safety climate subscale, item 1 had the lowest slope and was the least informative item, and item 7 had the highest slope and provided the highest amount of information. Items tend to provide the most information between −3 to 0 theta range. For the group-level safety climate subscale, item 11 had the lowest slope and was the least informative item, and item 6 had the highest slope and provided the highest amount of information. Items tend to offer the most information between −3 to 0 theta range.

**FIGURE 4:**
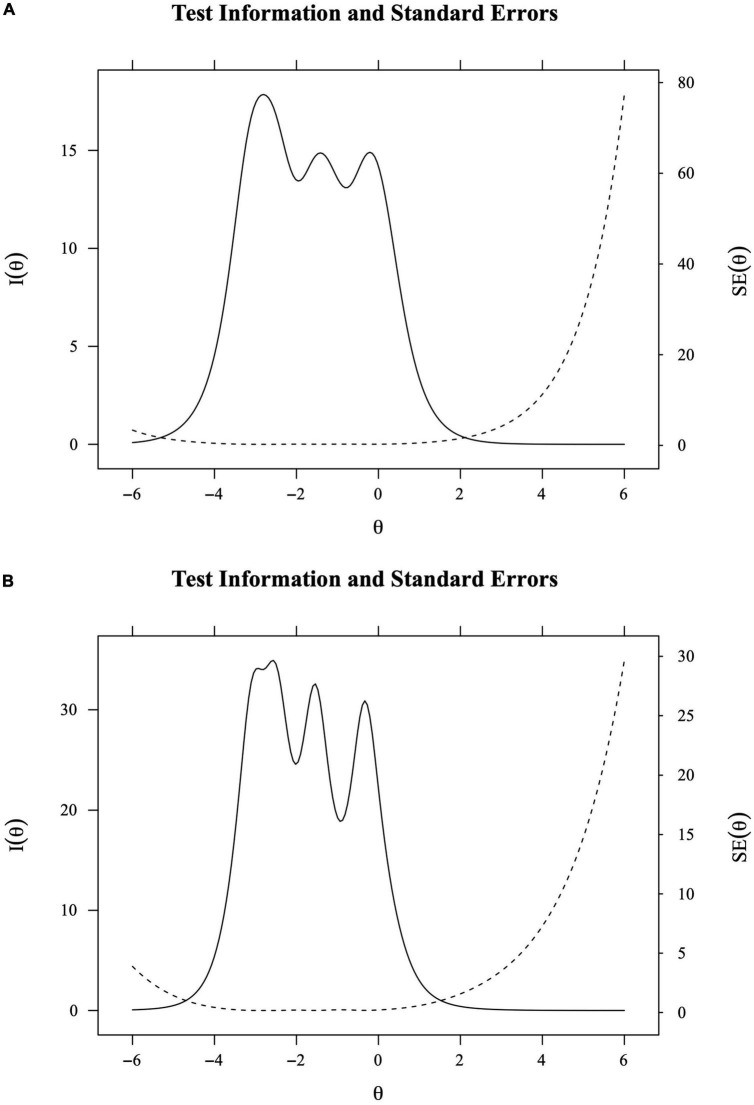
**(A)** Total test information function (aggregation of all the item information curves) for the Chinese organization-level safety climate subscale. **(B)** Total test information function (aggregation of all the item information curves) for the Chinese group-level safety climate subscale. *I* (θ) means information. *SE* (θ) means standard errors.

To reflect the sum of all item information and reflect how much information a test can provide at different latent trait levels based on the model, Figures 4A, B) show the total test information function for the Chinese organization- and group- level safety climate subscales, respectively.

### 3.3. Criterion related validity: Correlations between safety climate and safety-related variables

Results of the correlations between safety climate, safety behavior tendency, and safety rules attitude were presented in Table 6. As expected, the safety climate (at the total level, at the organization level, and at the group level) had significantly positive relationships with the safety behavior tendency, and safety rules attitude, all *p*’s < 0.001, indicating acceptable criterion related validity of the safety climate.

**TABLE 6:** Correlations of Chinese OSC and GSC with other measures (*N* = 1,570).

	OSC	GSC	Safety climate total	Safety behavior tendency	Safety rules attitude
OSC	–				
GSC	0.893***	–			
Safety climate total	0.965***	0.980***	–		
Safety behavior tendency	0.724***	0.774***	0.773***	–	
Safety rules attitude	0.354***	0.355***	0.364***	0.345***	–

OSC, organization-level safety climate; GSC, group-level safety climate. ***p < 0.001.

## 4. Discussion

The Chinese industry culture and the current political and economic situation required industries to pay more attention to the safety climate. Safety climate could help employees improve their happiness and industries improve their efficiency. For researchers, the studies on safety climate could contribute to digging into the connotations of the safety climate, factors that could impact the safety climate, and the possible mechanisms that related variables interacted with each other. However, in China, there was no well recognized scale to measure the safety climate. Therefore, the primary goal of the current study was to create the Chinese version of safety climate scale based on the English version of safety climate scale developed by Huang et al. (2017) which comprised 8 items for organization-level safety climate subscale and 11 items for group-level safety climate subscale, using an item response theory analytical approach. Since negative slope values in the 2-factor model could be considered problematic, the separate unidimensional models for the organization-level Chinese safety climate subscale and the group-level Chinese safety climate subscale were created. The assumption of unidimensionality and local independence were held. The indicators of reliability of the Chinese safety climate scale were good. Based on the IRT analyses performed in this study, items in the organization-level and group-level Chinese safety climate subscales had significantly different discrimination parameters, fitted well with the models, and had a substantive relationship with the latent traits. To be more specific, the discrimination parameters demonstrated that all items on the organization- and group- level safety climate subscales were capable of effectively discriminating between various levels of safety climate. Thus, the items could differentiate between participants who indicated high levels of safety climate as against those who reported low levels of safety climate. The overall organization- and group- level safety climate scale items, however, were more helpful in identifying respondents with low and average safety climate scores than those with high safety climate scores, according to the difficulty parameters.

The results further revealed that all Chinese safety climate scale items at the organization level fit the data based on the S-χ^2^ Item-level statistics, and the majority of the Chinese safety climate scale items at the group level fit the data based on the S-χ^2^ Item-level statistics, whereas items 6 and 7 appeared not to be a good fit based on the *p*-values. The likelihood ratio test indicated a good model-data fit. The Cronbach’s α and the McDonald’s omega of the Chinese organization- and group -level safety climate subscales showed good reliability. To sum up, the Chinese safety climate scale was reliable and valid to measure organization-level and group-level safety climates in China.

### 4.1. Strengths

The current study advances the researchers, industries, organizations, and safety professional groups in several ways. First, to our knowledge, the number of valid instrument for measuring safety climate that researchers can use in China is limited, especially when the measurement has to be separated into the organization level and group level. Ye et al. (2014) developed a 44-item Chinese safety climate scale with safety awareness, managerial commitment, supervisor behavior, safety policy, safety communication, safety training, risk preparedness dimensions. But it was not generally used in measuring safety climate in China. The reasons might be due to the relatively large number of items and dimensions. Mei et al. (2017) developed a 14-item Chinese safety climate scale with three dimensions, including safety attitudes, risk perception, and safety communication. However, Mei et al.’s (2017) safety climate scale only focused on the safety climate at the organization level. Therefore, the utility of safety climate evaluations in China can be more practical as a result of the development of the Chinese safety climate scale in this study. To be more specific, the application of the Chinese safety climate scale is beneficial for many researchers in China to explore the current characteristics, influencing factors, and underline mechanisms of safety climate. The lack of appropriate measurement instruments has stalled the localized research on safety climate in China. But fortunately, after the creation of the Chinese safety climate scale, investigators can have an accurate and valid tool to measure safety climate at the organization level and group level with the native language.

Second, the Chinese safety climate scale can be combined with other survey instruments, potentially increasing the likelihood of deepening our understanding of the linkages between safety climate and other variables. However, too many questions, which are measuring different aspects/variables, in a survey might make participants feel overwhelmed. Therefore, from the practical point of view, the Chinese safety climate scale with 8 items at the organization level and 11 items at the group level might at the balance point between a wider range of construct content and the efficiency of collecting multiple variables at once. Even, the Chinese safety climate scale created in this article was in the native language and it can be the ground for the theoretical development of safety climate based on the findings of subsequent studies conducted in China.

Third, it was anticipated that the Chinese safety climate scale would raise more awareness of the safety climate for industries, organizations, safety professional groups, and even the employees themselves in China. Along with researchers having the appropriate instruments to measure safety climate, they are able to obtain important and reasonable research conclusions, which can be introduced to highlight the importance of safety climate and subsequently advance safety development. And on the basis of the increased understanding of the safety climate and the importance that industries attach to it in China, on the one hand, the leadership of managers can be improved, the safety management actions can be promoted, on the other hand, employees can work in a safer environment, which is beneficial to employees’ physical and mental health, and then promote the work efficiency and increase industrial productivity with better safety assurance.

### 4.2. Limitations

The current study also has limitations. First, data were collected anonymously in this study, however, respondents might still have been influenced by the social desirability effect, which affects the accuracy of the results, such as most of the mean scores of items were relatively high (> 4) and the frequencies reported for response options 1 and 2 of the items were low. Anonymity is helpful to reduce the social desirability effect, however, participants still will give socially desirable answers even if their responses are anonymous (Patten, 2016). In this study, the link of the questionnaire was sent out by the department managers, which was similar to the way that employees received tasks assigned by the supervisors. In this way, employees might feel pressure to complete the questionnaire. Another reason that the social desirability effect might occur in this study is that Wenjuanxing is a very common platform used by researchers in China, and researchers are well aware of its anonymity, however, the employees who usually work in industries may not fully trust its anonymity. Other procedures, such as taking the survey in a relaxed environment and emphasizing the anonymity of participants, should be taken. In addition to the questionnaire method, a variety of techniques should be applied to measure the safety climate in the future. For example, indirect measures, such as implicit association tests can be used to evaluate individuals’ true thoughts about the safety climate to some extent.

Second, for the Chinese safety climate scale at the organization and group levels, Cronbach’s alpha values were higher than 0.9, which might indicate duplication of item content (Streiner, 2003; Panayides, 2013). Therefore, the Chinese safety climate scale in this article still have the potential to be shortened. Further studies need to be conducted to provide more evidence on whether to shorten the scale in the future.

Third, the Wenjuanxing links for the questionnaire were distributed to all workers by the department managers, therefore, workers with little time in the Shanghai Disney Resort industry had the opportunity to fill in the survey. However, those workers may have a limited view of the safety climate of the industry. Therefore, research data collected from those workers might distort the findings and conclusions of the study. Future studies should set clear criteria for workers who can participate in the study, such as participants should be workers who have worked in the industry for at least 3 months.

### 4.3. Implications for practice

The Chinese safety climate scale is important and it has some practical implications. For researchers, when they would like to measure the safety climate in China, they have an effective scale with localized language. In addition, the use of the Chinese safety climate in different situations or industries can be helpful to test its reliability and validity. Therefore, the results can be used to improve the scale, which can be used in a wider range of application scenarios and draw accurate conclusion. Furthermore, studies conducted with the Chinese safety climate scale are helpful to make recommendations for improving safety culture, identity possible safety-related issues, and find out potential area to make further investigation.

For companies, investigators can use the Chinese safety climate scale to conduct safety climate assessments for companies or organizations in China. After the measurement, data can be analyzed and then help to identify the current deficiencies, which can be corrected for the purpose of improving workers’ working environment, raising the awareness of safety, enhancing work efficiency and so on. The act of measuring the safety climate in Chinese companies can promote the importance of safety among employees. The frequency of the safety climate assessments can be varied on the basis of the size of the companies and the level of the risk involved in the manufacturing process.

## 5. Conclusion

In sum, this study developed the Chinese safety climate scale based on Huang et al.’s (2017) English version of safety climate scale. Two levels of safety climate, including the organization level and group level, were measured on the scale. Based on the nature of the two-level safety climate scale, a 2-factor IRT model was built to test the fitting of item and model. However, the values of the slope parameter were negative, which could be considered problematic. Therefore, the Chinese subscales of the organization-level and group-level safety climate were considered separately. In this study, the assumption of unidimensionality and local independence were held. The indicators of reliability of the Chinese safety climate scale were good. The confirmatory factor analysis (CFA) showed good model fit for a unidimensional model of the organization-level safety climate subscale, χ^2^(*df* = 20) = 129.158, *p* < 0.001, CFI = 0.994, TLI = 0.992, NFI = 0.993, IFI = 0.994, RMSEA = 0.059, 90% CI = (0.050, 0.069), and SRMR = 0.048. A unidimensional model also fits well for the group-level safety climate subscale, χ^2^(*df* = 44) = 219.727, *p* < 0.001, CFI = 0.996, TLI = 0.9925 NFI = 0.995, IFI = 0.996, RMSEA = 0.050, 90% CI = (0.044, 0.057), and SRMR = 0.046. Based on the IRT analyses performed in this study, items in the organization-level and group-level Chinese safety climate subscales had significantly different discrimination parameters, fitted well with the models, and had a substantive relationship with the latent traits. Results confirm that the Chinese safety climate scale can be applied as an effective instrument to measure the safety climate in China. This study revealed promising applicability of the Chinese safety climate scale as a reliable and valid tool to investigate the safety climate and together with other variables. Research using this Chinese safety climate scale can be contributed to the understanding of safety climate and increase awareness of the safety climate of industries in China.

## Data availability statement

The original contributions presented in this study are included in the article/supplementary material, further inquiries can be directed to the corresponding author.

## Ethics statement

The studies involving human participants were reviewed and approved by Nankai University. The patients/participants provided their written informed consent to participate in this study.

## Author contributions

All authors listed have made a substantial, direct, and intellectual contribution to the work, and approved it for publication.

## Footnotes

1
www.wjx.cn

## Appendix

**APPENDIX TABLE 1:** Safety climate scale.

Level	Item	English statement	Chinese statement
		Top management at this company:	这家公司的高层管理人员:
OSC	1	Tries to continually improve safety levels in each department.	各个部门尝试持续改进安全等级
	2	Quickly corrects any safety hazard (even if it’s costly).	迅速更正任何安全危害 (即使花费高昂)
	3	Requires each manager to help improve safety in his or her department.	要求每个经理协助改进其所在部门的安全
	4	Uses any available information to improve existing safety rules.	利用任何可用信息完善现有的安全规则
	5	Listens carefully to workers’ ideas about improving safety.	认真听取员工有关改进安全的意见
	6	Considers safety when setting production speed and schedules.	设定生产速度及进度时, 有将安全纳入考虑范围
	7	Provides workers with a lot of information on safety issues.	为员工提供大量有关安全问题的信息
	8	Gives safety personnel the power they need to do their job.	给予安全部门员工必要的权限以完成他们的工作
		My direct supervisor:	我的直接主管:
GSC	1	Frequently checks to see if we are all obeying the safety rules.	经常性地检查我们是否遵守安全规范
	2	Discusses how to improve safety with us.	与我们探讨如何改进安全
	3	Uses explanations (not just compliance) to get us to act safely.	合理地让我们安全行事 (而不只是一味服从)
	4	Emphasizes safety procedures when we are working under pressure.	当我们顶着压力工作时, 会强调安全规程
	5	Frequently tells us about the hazards in our work.	时常告诉我们日常工作中可能发生的危险
	6	Reminds workers who need reminders to work safely.	会提醒需要强调安全作业的员工
	7	Make sure we follow all the safety rules (not just the most importance ones).	确保我们遵守所有的安全规则 (而不仅仅是最重要的部分)
	8	Insists that we obey safety rules when fixing equipment or machines.	在修理设备或机器时, 坚决要求我们遵守所有的安全规则
	9	Is strict about safety at the end of shift, when we want to go home.	在我们轮班结束想回家时, 严格强调安全
	10	Spends time helping us learn to see problems before they arise.	会花一定时间协助我们学会预见安全问题
	11	Frequently talks about safety issues throughout the work week.	经常在工作时间内外与我们讨论安全问题

OSC, organization-level safety climate; GSC, group-level safety climate. The original items are from Huang et al. (2017). Likert 5 points (1 = completely disagree; 5 = completely agree). The Cronbach’s α of Chinese OSC and GSC is 0.912 and 0.937, respectively.

## References

[B1] Asad M. Kashif M. Sheikh U. A. Asif M. U. George S. Khan G. U. H. (2022). Synergetic effect of safety culture and safety climate on safety performance in SMEs: Does transformation leadership have a moderating role? *Int. J. Occup. Saf. Ergon.* 28 1858–1864. 10.1080/10803548.2021.1942657 34126869

[B2] Auzoult L. Ngueutsa R. (2019). Attitude to safety rules and reflexivity as determinants of safety climate. *J. Saf. Res.* 71 95–102. 10.1016/j.jsr.2019.09.016 31862049

[B3] Cantó-Cerdán M. Cacho-Martínez P. Lara-Lacárcel F. García-Muñoz Á (2021). Rasch analysis for development and reduction of symptom questionnaire for visual dysfunctions (SQVD). *Sci. Rep.* 11 1–10. 10.1038/s41598-021-94166-9 34290288 PMC8295373

[B4] Chalmers R. P. (2012). mirt: A multidimensional item response theory package for the R environment. *J. Statist. Soft.* 48 1–29. 10.18637/jss.v048.i06

[B5] Chiesi F. Lau C. Saklofske D. H. (2020). A revised short version of the compassionate love scale for humanity (CLS-H-SF): Evidence from item response theory analyses and validity testing. *BMC Psychol.* 8:20. 10.1186/s40359-020-0386-9 32087755 PMC7036195

[B6] Crocker L. Algina J. (1986). *Introduction to classical and modern test theory.* New York, NY: Harcourt Brace Jovanovich.

[B7] Embretson S. E. Reise S. P. (2013). *Item response theory.* London: Psychology Press.

[B8] Huang Y. H. Lee J. Chen Z. Perry M. Cheung J. H. Wang M. (2017). An item-response theory approach to safety climate measurement: The liberty mutual safety climate short scales. *Accid. Anal. Prev.* 103 96–104. 10.1016/j.aap.2017.03.015 28391093

[B9] JASP Team (2022). *JASP (version 0.16.3) [computer software].* Available online at: https://jasp-stats.org/ (accessed July 16, 2022).

[B10] Kao K.-Y. Thomas C. L. Spitzmueller C. Huang Y. (2021). Being present in enhancing safety: Examining the effects of workplace mindfulness, safety behaviors, and safety climate on safety outcomes. *J. Bus. Psychol.* 36 1–15. 10.1007/s10869-019-09658-3

[B11] Kim H. Hong J. Cho I.-K. Lee D. Cho E. Jun J. Y. (2022). Psychometric properties of the stress and anxiety to viral epidemics-9 scale among frontline nursing professionals working in the COVID-19 inpatients ward. *Front. Psychiatry* 13:934202. 10.3389/fpsyt.2022.934202 35935440 PMC9353028

[B12] Kim N. K. Rahim N. F. A. Iranmanesh M. Foroughi B. (2019). The role of the safety climate in the successful implementation of safety management systems. *Saf. Sci.* 118 48–56. 10.1016/j.ssci.2019.05.008

[B13] Li N. Sun Z. Niu L. (2018). Pursue and trace of the effects of the different types of safety awareness on the safety behaviors of the coal miners. *J. Saf. Environ.* 18 1863–1867.

[B14] Li Y. Xu Y. Wang M. Xue J. Feng Y. Xu Y. (2023). Safety management actions improvement as leverage for safety climate promotion in high-risk industris. *Adv. Psychol. Sci.* 31 33–34. 10.3724/sp.j.1042.2023.00033

[B15] Luo T. (2020). Safety climate: Current status of the research and future prospects. *J. Saf. Sci. Resil.* 1 106–119. 10.1016/j.jnlssr.2020.09.001

[B16] Mei Q. Zhang C. Li W. Liu S. (2017). Relationship among safety culture, safety climate and staff’s safety behavior: An empirical analysis of SMEs in high-risk industry. *J. Syst. Manag.* 26 277–286.

[B17] Newaz M. T. Davis P. Jefferies M. Pillay M. (2019). The psychological contract: A missing link between safety climate and safety behaviour on construction sites. *Saf. Sci.* 112 9–17. 10.1016/j.ssci.2018.10.002 30876525

[B18] Omidi L. Salehi V. Zakerian S. Nasl Saraji J. (2022). Assessing the influence of safety climate-related factors on safety performance using an integrated entropy-TOPSIS approach. *J. Ind. Prod. Eng.* 39 73–82. 10.1080/21681015.2021.1958937

[B19] Panayides P. (2013). Coefficient alpha: Interpret with caution. *Eur. J. Psychol.* 9 687–696. 10.5964/ejop.v9i4.653 33680184

[B20] Patten M. (2016). *Questionnaire research: A practical guide*, 4th Edn. Milton Park: Taylor & Francis Group.

[B21] Ricci F. Panari C. Pelosi A. (2022). Safety compliance in a sample of Italian mechanical companies: The role of knowledge and safety climate. *Eur. J. Investig. Health Psychol. Educ.* 12 281–294. 10.3390/ejihpe12030020 35323206 PMC8947296

[B22] Rizopoulos D. (2007). LTM: An R package for latent variable modeling and item response analysis. *J. Statist. Softw.* 17 1–25. 10.18637/jss.v017.i05

[B23] Samejima F. (2016). “Graded response models,” in *Handbook of item response theory*, Vol. one ed. van der Linden W. J. (New York, NY: Chapman and Hall/CRC), 123–136.

[B24] Srem-Sai M. Quansah F. Hagan J. E. Jr. Ankomah F. Frimpong J. B. Ogum P. N. (2022). Re-assessing the psychometric properties of stress appraisal measure in Ghana using multidimensional graded response model. *Front. Psychol.* 13:856217. 10.3389/fpsyg.2022.856217 35664186 PMC9161214

[B25] Streiner D. L. (2003). Starting at the beginning: An introduction to coefficient alpha and internal consistency. *J. Pers. Assess.* 80 99–103. 10.1207/S15327752JPA8001_18 12584072

[B26] Taber K. S. (2018). The use of Cronbach’s alpha when developing and reporting research instruments in science education. *Res. Sci. Educ.* 48 1273–1296. 10.1007/s11165-016-9602-2

[B27] Toland M. D. (2014). Practical guide to conducting an item response theory analysis. *J. Early Adolesc.* 34 120–151. 10.1177/0272431613511332

[B28] Vilca L. W. Chambi-Mamani E. L. Quispe-Kana E. D. Hernández-López M. Caycho-Rodríguez T. (2022). Functioning of the EROS-R scale in a clinical sample of psychiatric patients: New psychometric evidence from the classical test theory and the item response theory. *Int. J. Environ. Res. Public Health* 19:10062. 10.3390/ijerph191610062 36011696 PMC9407833

[B29] Ye X. Li X. Wang Z. (2014). Safety climate and safety behavior: The mediating role of psychological capital. *Soft Sci.* 28 86–90. 10.13956/j.ss.2014.01.002

[B30] Ye Z. Liang M. Zhang H. Li P. Ouyang X. R. Yu Y. (2018). Psychometric properties of the Chinese version of resilience scale specific to cancer: An item response theory analysis. *Qual. Life Res.* 27 1635–1645. 10.1007/s11136-018-1835-2 29569015

[B31] Zhao L. Lyu X. Jiang H. Gao X. (2022). Musicokinetic and exercise therapies decrease the depression level of elderly patients undergoing post-stroke rehabilitation: The moderating effect of health regulatory focus. *Front. Psychol.* 13:889510. 10.3389/fpsyg.2022.889510 36046420 PMC9421369

[B32] Zohar D. (1980). Safety climate in industrial organizations: Theoretical and applied implications. *J. Appl. Psychol.* 65 96–102. 10.1037/0021-9010.65.1.96 7364709

[B33] Zohar D. (2000). A group-level model of safety climate: Testing the effect of group climate on microaccidents in manufacturing jobs. *J. Appl. Psychol.* 85 587–596. 10.1037/0021-9010.85.4.587 10948803

[B34] Zohar D. Luria G. (2005). A multilevel model of safety climate: Cross-level relationships between organization and group-level climates. *J. Appl. Psychol.* 90 616–628. 10.1037/0021-9010.90.4.616 16060782

